# A protocol for rapid monocyte isolation and generation of singular human monocyte-derived dendritic cells

**DOI:** 10.1371/journal.pone.0231132

**Published:** 2020-04-09

**Authors:** Thaize Quiroga Chometon, Mariana da Silva Siqueira, Julie Carmo Sant´anna, Matheus Rogério Almeida, Mariana Gandini, Ana Cristina Martins de Almeida Nogueira, Paulo Renato Zuquim Antas

**Affiliations:** 1 Clinical Immunology Laboratory, Oswaldo Cruz Institute, FIOCRUZ, Rio de Janeiro, Brazil; 2 Post-Graduation Program in Sanitary Surveillance, National Institute of Quality Control in Health, FIOCRUZ, Rio de Janeiro, Brazil; 3 Viral Immunology Laboratory, Oswaldo Cruz Institute, FIOCRUZ, Rio de Janeiro, Brazil; Universidade de Sao Paulo Instituto de Quimica, BRAZIL

## Abstract

The monocyte-derived dendritic cells (moDCs) are a subset of dendritic cells widely used in immunological studies as a convenient and easy approach after isolation of mononuclear cells directly from peripheral blood mononuclear cells (PBMC). Both the purification and cell culture of monocytes impact on the differentiation of monocytes into moDCs. The methodology to isolate and differentiate monocytes into moDCs is still controversial. We aimed to compare three different protocols for monocyte isolation from PBMC: 1) Cold-aggregation; 2) Percoll gradient; and 3) Magnetic beads cell-enrichment. Additionally we also compared four different monocyte differentiation and culture techniques: 1) Cell culture media; 2) Serum sources; 3) required GM-CSF and IL-4 concentrations; 4) Cell culture systems. We used flow cytometry analysis of light scattering and/or expression of pan surface markers, such as CD3, CD14 and CD209 to determine isolation/differentiation degree. Purified PBMC followed by two steps of cold aggregation, yielded cell viability around 95% with poor monocyte enrichment (monocytes increase vs. lymphocytes reduction was not statistically significant, p>0.05). Conversely, monocyte isolation from PBMC with discontinuous Percoll gradient generated around 50% cell viability. Albeit, we observed a significant reduction (p≤0.05) of lymphocytes contaminants. The magnetic beads cell-enrichment yield cell viability higher than 95%, as high as a significant lymphocyte depletion (p≤0.005) when compared to all other techniques employed. The moDCs showed better differentiation based on increased CD209 expression, but lower CD14 levels, when cells were cultured in RPMI medium plus 500IU/mL of both GM-CSF and IL-4 in a semi-adherent fashion. Serum sources showed no influence on the culture performance. In conclusion, the magnetic beads cell-enrichment showed superior cell viability, indicating that this approach is a better choice to isolate monocytes, and moDCs cultured afterwards in appropriate medium, serum, cytokines and culture system might influence the monocytes differentiation into moDC.

## 1. Introduction

Monocyte-derived dendritic cells (moDCs) are a subset of Dendritic Cells (DCs) widely used in immunological studies as a convenient and easy approach after isolation of mononuclear cells directly from circulation. Human moDCs can be generated *in vitro* from peripheral blood CD14^+^ monocytes or from CD34^+^ progenitors [1].

DCs are highly motile immune cells, ubiquitously scattered throughout tissues, which represent a heterogeneous group of cells sharing the same function. They continuously sample the environment for antigens by means of endocytosis, owing to their high phagocytic activity and antigen processing capacity [2, 3]. *In vivo*, moDCs are present only under inflammatory conditions, and co-localize with any invading pathogen. This is in contrast to conventional DCs that are present in normal steady state [3]. The generation and use of moDCs has been extensively described in both basic and applied research, such as in the development of cancer immunotherapies. They are considered the gold standard for DC-associated *in vitro* studies [4]. Classically, monocyte differentiation into moDCs is facilitated by *in vitro* supplementation of granulocyte-macrophage colony-stimulating factor (GM-CSF) and interleukin (IL)-4, to generate immature DCs. Typically, a key phenotypic change between monocyte and moDCs is the resilient loss of CD14 expression (CD14^low/-^), with concomitant increase in CD209 expression [5].

In fact, moDCs have been used in clinical approaches with moderately encouraging results [6, 7]. However, recent studies have shown that monocyte purification methods, by means of both flask adherence and magnetic sorting, led to different phenotypic and functional characteristics of the DCs yielded [1, 2]. In addition, the culture medium used may hinder the differentiation of DCs. Essentially, any procedure to isolate monocytes *in vitro* may have an impact on the subsequent DCs function, probably affecting both the ability to produce cytokines and T-cell interactions [2].

The use of different combinations of cytokines, growth factors and adjuvants could be used for the differentiation and maturation of DCs. Differences in their compositions, concentration as well as in time and duration of stimulation could generate cells with into different phenotypes and consequently cells with different immunological and tolerogenic potentials [8, 9]. For example, long culturing processes may negatively affect the function of DCs by generating less immunogenic cells [8].

There are virtually no comparative *in vitro* analyses addressing different protocols for generating moDC. Therefore, in this study we assessed the distinct *in vitro* techniques focusing on: 1) monocyte enrichment procedures, especially in-house cold-aggregation, and both commercially available Percoll and immune-magnetic beads; 2) cell culture minimal medium testing, such as RPMI-1640 or DMEM; 3) supplementation features, using inactivated fetal calf (FBS) or AB human serum (HS); 4) required stimulatory cytokine concentration, using rhGM-CSF and rhIL-4; and 5) cell culture systems, specifically round-bottom polystyrene tubes or flat-bottomed tissue culture plates. The read out for differentiation was phenotypic characterization by flow cytometry, in order to demonstrate the feasibility of the proposed protocol for generating canonical moDC.

## 2. Materials and methods

### 2.1. Source of human mononuclear cells

Mononuclear cells were freshly isolated from leukocyte concentrate (buffy-coat) collected from healthy donor volunteers (n = 26) enrolled at the Hematology Unit of the Clementino Fraga Filho University Hospital (Federal University of Rio de Janeiro-UFRJ). Ethical clearance for the use of human subjects was obtained from the designated health facility. Written informed consent was obtained from each person after receiving information about use of their blood samples. The biological material was anonymized. The study was approved by both local and Fiocruz IRBs and could be found in the HUCFF (http://www.hucff.ufrj.br/pesquisa/cep) and the IOC-Fiocruz (protocol # 35775014.0.0000.5248; https://portal.fiocruz.br/comites-de-etica) web addresses for the institutional review boards that approved the study procedures.

### 2.2. Isolation of human Peripheral Blood Mononuclear Cells (PBMC) and purification of monocytes

PBMC were separated (> 92% purity) from the buffy coat within 24 hours after obtaining the blood specimens from all study participants using a density gradient (Ficoll-Hypaque, Sigma-Aldrich Inc., USA), following a protocol as previously described [10]. Subsequently, in order to optimize the monocyte isolation method, we isolated the target cells using three different strategies in parallel, as described below.

#### 2.2.1. Cold-aggregation

It has been observed that monocytes spontaneously and rapidly aggregate *in vitro* at 4°C, called cold-aggregation purification procedure [5]. Therefore, monocytes were segregated from lymphocytes by resuspending the PBMCs in RPMI-1640 medium (Sigma Immunochemicals, USA) supplemented with 10% fetal bovine serum (FBS, Cultilab, Brazil) and incubating for 30 min at 4°C with continuous agitation. Monocytes then spontaneously sedimented [5], and two successive rounds of cold-aggregation were subsequently performed. We determined cell viability by trypan blue dye. The monocyte population was determined by flow cytometry based on forward and side scatter (FSC *vs*. SSC) gating, as well as on the expression or lack of the surface markers anti-hCD14-FITC (Dilution 1:100; Clone M5E2; BioLegend, USA; cat 982502) and anti-hCD3-Alexa 647 (Dilution 1:100; Clone OKT3; eBiosciences, USA; cat 51-0037-73).

#### 2.2.2. Cold-aggregation and self-generating discontinuous density gradient

The monocyte isolation procedure was prepared in two sequential steps: [1] cold-aggregation through the protocol by Santos et al [5], followed by [2] a discontinuous density gradient (Percoll, Sigma-Aldrich Inc., USA) adapted from Repnik et al [11]. Cell viability and purity were then determined as described above.

#### 2.2.3. Immunomagnetic purging of lymphocytes

The monocyte population was enriched by negative selection of unlabeled target cells using a human monocyte enrichment kit (EasySep, Stemcell Tech., France), according to the manufacturer´s protocol. To obtain a high degree of monocyte purification, the commercial kit contains a combination of human monoclonal antibodies bound in bispecific tetrameric antibody complexes, which are directed against cell surface antigens on human blood cells (CD2, CD3, CD19, CD20, CD56, CD66b, CD123, glycophorin A) and dextran. Therefore, purified PBMC (50 to 150 × 10^6^ cells) were subjected to magnetic separation using antibody-coated microbeads targeting unwanted populations. PBMCs were eluted with HBSS buffer (Ca^++^ and Mg^++^ free; Gibco BRL, Invitrogen Inc., USA) containing 2% human AB serum (HS, Sigma-Aldrich Inc., USA) and 1 mM EDTA (Gibco BRL, Invitrogen Inc., USA). The unbound cells (monocytes) were then collected. The time required to complete this negative depletion was less than one hour. The purity of the monocyte population was evaluated by flow cytometry based on FSC and SSC features.

For comparative purposes only, a subset of experiments was performed by means of positive selection of CD14-labeled target cells using a human magnetic antibody cell sorting (MACS) system (Miltenyi Biotec, Bergisch-Gladbach, Germany), according to the manufacturer´s procedure. Both time required (roughly one hour) and purity of the monocyte population were determined as described for the negative selection procedure.

### 2.3. Complete Culture Media (CCM) evaluation in differentiation of moDCs

After purification, cell density was adjusted to 0.5 × 10^6^ cells/mL. Cells were cultured in 24-well tissue culture plates (Falcon Inc., USA), resuspended in either RPMI-1640 or DMEM culture medium (Sigma Immunochemicals, USA) supplemented with either 10% FBS or 10% HS. moDC differentiation medium (CCM) was obtained by supplementing each culture media described above with 300 or 500 UI/mL of recombinant human (rh) GM-CSF and 300 or 500 UI/mL of rhIL-4 (PeproTech Inc., USA). After 48 hours, half of the total volume of each well was carefully removed, and the respective CCM was added for an additional 2 days. Non differentiated monocytes (negative control) were set up in parallel wells and received the respective media without cytokines. Based on a prior study, macrophages were generated at the onset of the differentiation timeline [12].

### 2.4. Cytokine concentration and cell culture system evaluations

After purification, 0.5 × 10^6^ cells/mL were cultured into either 24-well flat-bottomed tissue culture plates (Falcon Inc., USA) or round-bottomed polypropylene tubes (Falcon Inc., USA), in RPMI-1640 culture medium supplemented with 10% HS. For differentiation of monocytes into moDCs, the culture medium was supplemented with 300 or 500 UI/mL of hrGM-CSF and 300 or 500 UI/mL hrIL-4. Both media replacements and control (baseline) cultures were set up as described earlier. In tissue culture plates, cells were detached and collected using a 5 mL syringe rubber plunger in gentle, one-way movements, followed by washing the wells with ice-cold PBS pH 7.4 and gently transferring cells into round-bottomed polypropylene tubes (Falcon Inc., USA) for staining procedure. Samples were stained with the following mAbs: anti-hCD14-PE/Dazzle 594 (Dilution 1:100; Clone HCD14; BioLegend, USA; cat 325634) and anti-hCD209-PE (Dilution 1:200; Clone 9E9A8; BioLegend, USA; cat 330106). The precise staining procedure and flow cytometer analysis performed are described in the following section.

### 2.5. Analysis of monocyte differentiation and moDC generation

Cell viability was promptly evaluated by trypan blue exclusion. Phenotyping for cell differentiation and generation were performed by flow cytometry using scatter parameters and/or pan surface markers, such as CD3 (T-lymphocyte marker), CD14 (monocyte marker), and CD209 (moDCs marker).

In the flow cytometry procedures, specific commercial antibodies were diluted in FACS buffer (PBS in 1% BSA and 0.05% NaN3). Then, human PBMCs, isolated monocytes, non-differentiated monocytes, and moDCs were incubated for 30 min at 4°C. The cells were then washed twice in ice-cold FACS buffer and acquisitions were performed on a CyAn ADP Analyzer (Beckman Coulter, USA) for monocyte analysis and a FACSAria (BD Biosciences, USA) for non-differentiated monocytes and moDCs evaluation. Warranty of the stability of acquisition was verified for every experiment using a dotplot Time *vs*. SSC-H or fluorescence.

The following lasers and filters were used: At 488 nm laser the cyan 530/40 filter was used for FITC detection, Cyan 575/25 and FACSAria 576/26 filters were used for PE detection, Cyan 613/20 and FACSAria 610/20 filters were used for PE-Dazzle594 detection. At 635 nm laser cyan 665/20 was used for AlexaFluor 647 detection.

**Fig 1:**
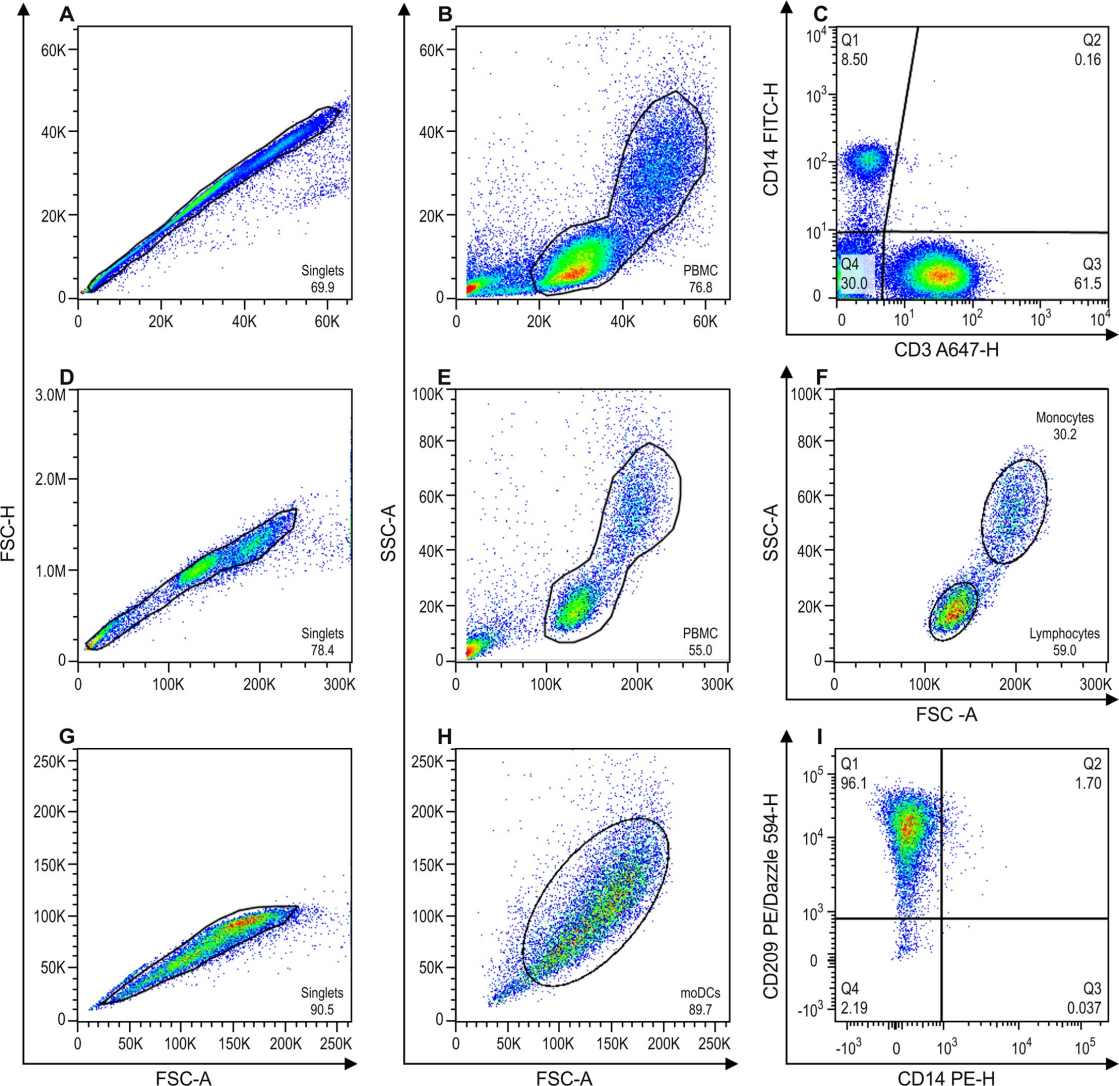
Gate strategy for flow cytometry analysis. A, B and C = representative experiment after ficoll gradient followed by two steps of cold-aggregation and by cold-aggregation + percoll gradient for isolation of monocytes. D, E and F = representative experiment after ficoll gradient followed by Immunomagnetic beads for isolation of monocytes. G, H and I = representative experiment for monocyte differentiation to moDC. A = Dotplot of size FSC-A *vs* FSC-H for doublets exclusion, gated on singlets. B = Dotplot of FSC-A *vs* SSC-A for PBMCs, inside singlets gate. C = Dotplot of CD14 *vs* CD3, inside PBMC gate, where Q1 represents monocytes (CD14+) and Q3 represents T lymphocytes (CD3+). D = Dotplot of FSC-A *vs* FSC-H for doublets exclusion, gated on singlets. E = Dotplot of FSC-A *vs* SSC-A for PBMCs, inside singlets gate. F = Dotplot of FSC-A *vs* SSC-A inside the PBMC gate, where lymphocytes and monocytes were gated according to their size and granulosity characteristics. G = Dotplot FSC-A *vs* FSC-H for doublets exclusion of moDC after the differentiation protocol, gated on singlets. H = Dotplot of FSC-A *vs* SSC-A for moDCs, inside singlets gate. I = Dotplot of CD14 *vs* CD209, inside moDCs gate, where Q1 represents moDCs (CD209+) and Q3 represents non-differentiated monocytes (CD14+). All percentage and statistical analysis were performed inside the PBMC gate, thus excluding cell debris.

The gate strategies were based on: doublets exclusion with singlet gate in FSC-H and FSC-A dotplot (Fig 1A, 1D and 1G) and from singlet gate dotplot size (FSC)-A *vs* granularity (SSC)-A used to exclude cellular debris (Fig 1B, 1E and 1H). In addition, to determine monocyte differentiation we analyzed the frequency of monocyte and lymphocyte populations inside the PBMC gate (Fig 1B and 1E). We evaluated cold aggregation and self-generating discontinuous density gradient (Percoll) monocytes isolation technique by verifying the expression of CD3 and CD14 inside PBMC gate (Fig 1C). The inference of the immunomagnetic monocytes population purification procedure was performed by analyzing monocyte and lymphocyte populations in two distinct gates (Fig 1F). To analyze moDCs differentiation, after the doublets exclusion (Fig 1G), we determined a gate on the population of interested in the dotplot size (FSC)-A *vs* granularity (SSC)-A (Fig 1H) and evaluated the percentage of cells positive for CD209 and CD14 (Fig 1I).

In addition, LIVE/DEAD^™^ Fixable Green Dead Cell Stain Kit (Dilution:1:1000; Life Technologies, lot 177445, cat L34969) viability test was used, in accordance with the manufacture recommendation. Viable cells (LIVE/DEAD^-^ events) were gated one week after culture to compare moDCs differentiation in tubes and plates.

### 2.6. LPS stimulation

In order to verify whether the differentiation of moDCs lead to a functional feasible DCs, we performed a response/maturation moDCs assay, based on the method described by Elkord et al. [2]. Herein, we used unstimulated DCs (immature DCs) as well as DCs stimulated with Lipopolysaccharide (LPS) from Escherichia coli (026: B6) at 0.5 μg/ml (Sigma-Aldrich) for 24 hours. After LPS stimulation the cells cultures were incubated at 37°C and 5% CO_2_ for 24 hours.

Subsequently, cells were removed from plates, washed in PBS pH 7.4, transferred into tubes (Falcon Inc., USA) and stained for 20 min in the dark at 4°C with anti-CD86 APC (Dilution 1:100; Clone IT2.2; BioLegend, USA; cat 305412) and anti-CD80 APC Alexa 750 (Dilution 1:40; Clone HA5.2B7; Beckman Coulter, USA; cat PNB30643). Anti-CD14 PE-Dazzele 594 and anti-CD209 PE were used for phenotyping monocytes and moDCs respectively. For staining and analysis procedures, we used the same protocol as described in section 2.5. Regarding fluorescence detection, the following lasers and filters were used: At 488 laser FACSAria 576/26 filter was used for PE detection, FACSAria 610/20 filter was used for PE-Dazzle594 detection. At 635 laser FACSAria 660/20 filter was used for APC detection and FACSAria 780/60 filter was used for APC- AlexaFluor 750 detection.

### 2.7. Data analyses and statistical evaluation

The FlowJo software version 10.0 (TreeStar Inc., USA) was used for all cytometric data analyses. Data is expressed as mean ± SD or as individual values and evaluated with GraphPad prism software v5.1 (La Jolla, USA) using non-parametric statistical tests. Differences between analyzed groups (immunophenotyping) were determined using Mann-Whitney test and Wilcoxon matched-pairs signed-ranks test with *p ≤ 0.05.

## 3. Results

First, to obtain blood monocytes, we compared three cell-isolation protocols: cold aggregation, cold aggregation/Percoll gradient, and magnetic beads. In order to verify target cell isolation yields, we analyzed target cell frequency both by morphological features (FSC *vs*. SSC), and expression of a specific monocyte marker (CD14) by flow cytometry.

We then compared four different cell culture and differentiation techniques: 1) cell culture media, 2) supplemented serum sources, 3) GM-CSF and IL-4 concentrations, and 4) cell culture systems. We used flow cytometry analysis of light scattering (FSC *vs*. SSC) and/or expression of pan surface markers (CD3, CD14 and CD209) to determine the degree of isolation/differentiation.

Monocytes, usually enriched from PBMCs, are commonly used to generate myeloid DCs *in vitro*. Depending on the method applied, the efficiency of the enrichment and generation of these cells could vary [1, 5, 13]. Here, we performed monocyte isolation from PBMC using a two-step protocol. First, (step a) monocytes were enriched by a one round of cold aggregation followed by re-purification of monocytes either by (step b) a second round of cold aggregation or (step c) a self-generating discontinuous Percoll gradient technique.

To reduce the cost, labor, and any possible loss of potential moDC precursors from the PBMCs, we primarily determined the performance of the distinct segregation techniques. This was done through assessment of the possible impact on monocyte viability and purity by trypan blue exclusion and the expression of monocyte and T lymphocyte pan surface markers, CD14 and CD3, respectively.

**Fig 2:**
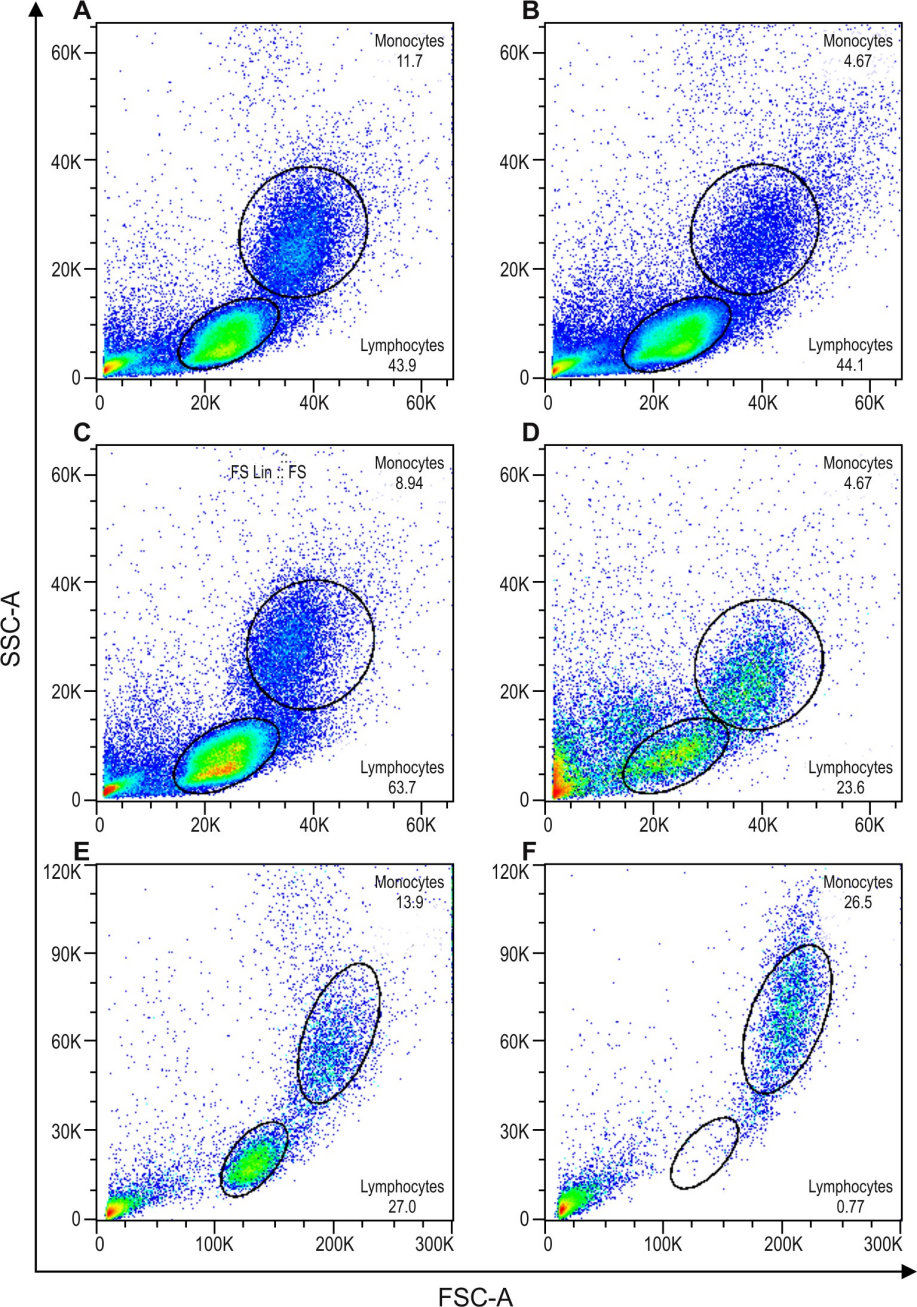
Flow cytometry light scattering (forward scatter, FSC, size *vs*. side scatter, SSC, granularity) demonstrating a typical profile from a representative experiment. A, C, and E = Dotplots after PBMC isolation, and the respective subsequent dot plots; B = two consecutive steps of in house cold aggregation (n = 3), D = in house cold aggregation plus self-generating discontinuous Percoll gradient (n = 6), and F = magnetic beads cell-enrichment (n = 10). Percentage of cells as follows: (A) Monocytes 11.7% and lymphocytes 43.9%; (B) Monocytes 4.67% and lymphocytes 44.1%; (C) Monocytes 8.94% and lymphocytes 63.7%; (D) Monocytes 20.6% and lymphocytes 23.6%; (E) Monocytes 14.9% and lymphocytes 26.7%; (F) Monocytes 27.7% and lymphocytes 0.6%.

Notably, after purification step (a), only negligible improvements in monocyte frequency were obtained, especially the decrease of CD3 (CD3 after step (a) 56.8 ±9.3% *vs*. in PBMC 63.8 ± 9.9% and CD14 after step (a) 16.1± 6.2% *vs* 5.8 ± 1.2%). After the second cold aggregation round, or protocols steps (a) + (b), we observed cell viability higher than 95%. However, monocyte frequency enrichment was unsatisfactory, as evidenced by the slight decrease of monocytes, and unchanged lymphocyte rates (Fig 2A and 2B, S1 Table). This was further confirmed when we examined the percentage of CD14^+^ cells obtained in the PBMC (5.8 ± 1.2%), and after steps (a) + (b) (4.6 ± 0.7%). In addition, we observed similar CD3 expression after steps (a) + (b), compared to PBMC (63.8 ± 9.9% *vs*. 62.1 ± 4.2%, respectively). Taken together, although enhanced cell viability was obtained, the two rounds of cold aggregation did not constitute an appropriate procedure for monocyte enrichment because of the reduced monocyte final yield, at least under our experimental conditions.

In the enrichment protocol using the cold-aggregation method followed by self-generating discontinuous Percoll gradient, or step protocols (a) + (c), we detected a reduction of roughly 50% in cell viability as determined by trypan blue and by an increase of events outside lymphocytes and monocytes gates (Fig 2C and 2D). Furthermore, we observed a critical lymphocyte reduction, concomitant with an increased monocyte frequency. A change in the scatter feature of cells was observed when comparing step protocols (a) + (b) (Fig 2C and 2D) with (a) + (c) (Fig 2E and 2F). The rate of CD14^+^ cells after step protocols (a) + (c) was considerably higher compared to PBMC (48.4 ± 17.7% *vs*. 6.8 ± 2.2%, respectively). Moreover, the CD3^+^ cells showed a significant decrease (p-level ≤ 0.05) after step protocols (a) + (c) when compared to PBMC (21.7 ± 10.8% *vs*. 61.7 ± 5.3%, respectively), thus demonstrating that cold aggregation plus Percoll gradient steps significantly diminished lymphocytes contamination (S1 Table).

Although the Percoll procedure yielded superior monocyte enrichment, there was a serious loss in cell viability. These results suggest that either the lengthy process or the toxicity of the Percoll reagent might lead to increased cell death [14].

A third technique that we investigated was the magnetic beads cell-enrichment technique. This was performed immediately after PBMC purification, and avoiding cold aggregation. In order to evaluate the purity of the monocyte population obtained, we compared the cell populations based only on the forward and side scatter characteristics. We observed a significant depletion (p ≤ 0.005) of lymphocytes compared to PBMC (8.3 ± 5.2% *vs*. 75.1 ± 8.5%). In addition, we detected highly significant monocyte enrichment (p ≤ 0.005) after magnetic isolation (Fig 2F) when compared to the protocol following step (a) only (89.0 ± 5.1% vs. 22.7 ± 8.6%) (Fig 2E and 2F).

Magnetic beads monocyte enrichment was the method of choice for subsequent differentiation of moDCs, since it has proven to reach better purification results when compared to the two others techniques (S1 Table).

**Fig 3:**
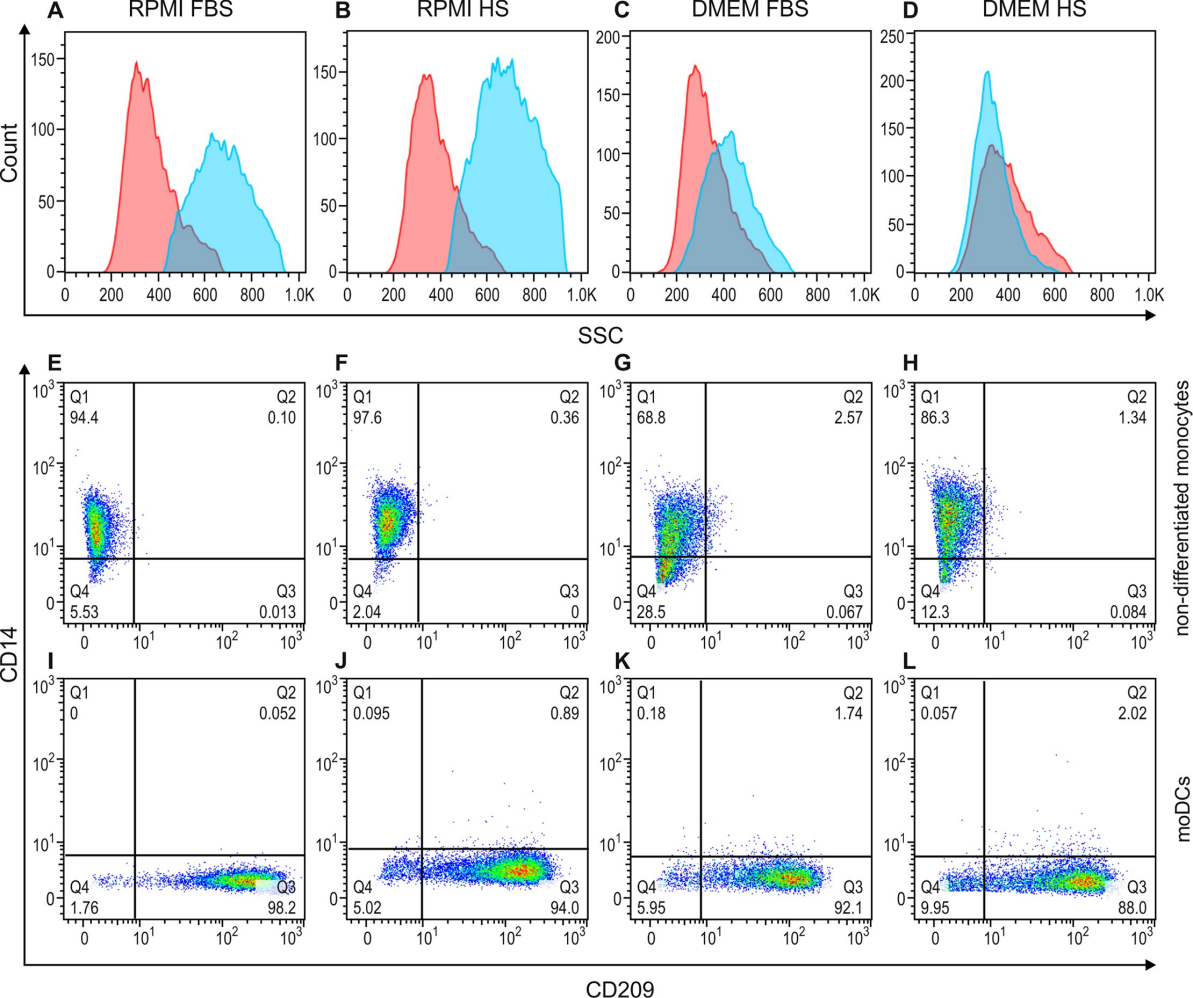
Flow cytometry overlay histograms for side scatter (A-D) and dot plots for CD14 and CD209 staining (E-L) demonstrating typical cell profiles from a representative experiment out of three. Side scatter (SSC, granularity) in non-differentiated monocytes (red shaded histogram) and differentiated moDCs (blue shaded histogram) cultured in either RPMI (A and B) or DMEM medium (C and D), supplemented with either FBS (A and C) or HS (B and D). CD14 and CD209 surface marker expression on non-differentiated monocytes (E-H) and moDCs (I-L) cultured in either RPMI (E, F, I and J) or DMEM medium (G, H, K and L), supplemented with either FBS (E, G, I and K) or HS (F, H, J and L). Cells were stained with CD14-FITC (y-axis) and CD209-PE (x-axis). The percentages of cells are indicated in each quadrant.

Having established a protocol for obtaining purified monocytes directly from PBMCs using magnetic beads cell-enrichment, our next objective was to determine the best method for moDC generation. Again, flow cytometry tools were used to distinguish between differentiated (moDCs, Fig 3A–3D blue shaded histogram and 3I-3L) and non-differentiated monocytes (Fig 3A–3D red shaded histogram and 3E-3H).

The moDCs cultivated in RPMI (Fig 3A and 3B) displayed typical scatter characteristics, and were as abundant as granulated cells, compared to those cultivated in DMEM (Fig 3C and 3D) as well as non-differentiated monocytes (Fig 3A–3D). There were no significant differences in CD14 and CD209 expression on cells cultivated with either RPMI or DMEM (Fig 3E–3L). Based on the scatter parameters of cells cultivated in RPMI, subsequent experiments were performed using RPMI medium.

We next tested the source of commercial serum, either HS or FBS, and observed similar results for cell culture and differentiation experiments. We determined to supplement the RPMI medium with HS since no differences were observed and we were working with human cells (Fig 3B, 3F, 3J; and 3A, 3E, 3I for HS and FBS, respectively).

**Fig 4:**
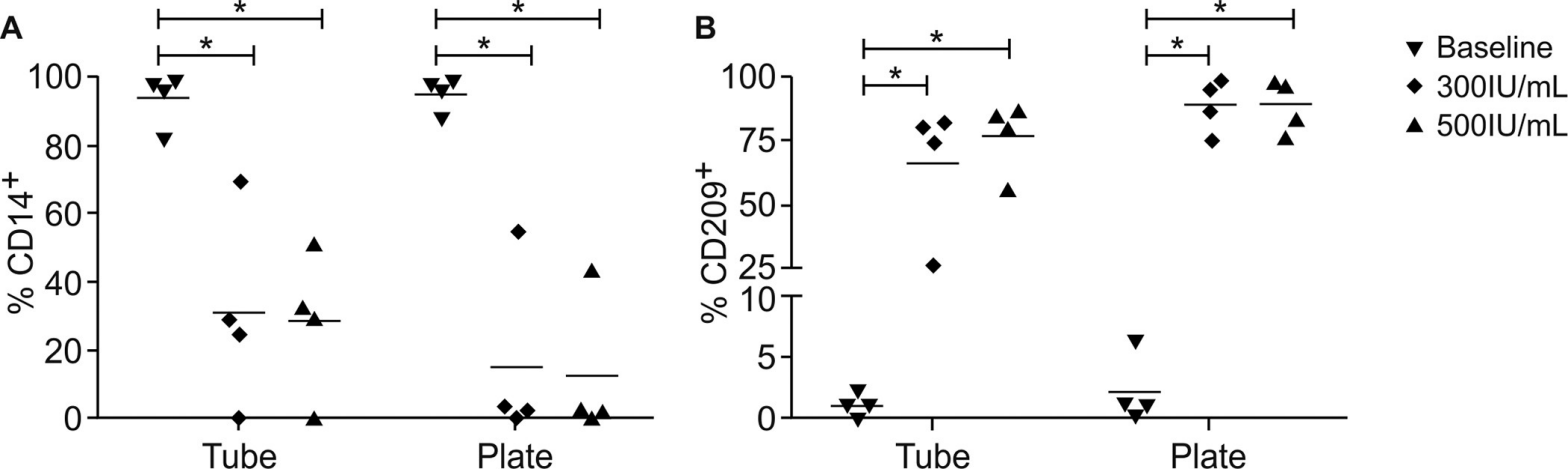
Comparison of the percentage of moDCs or non-differentiated monocytes expressing CD14 or CD209. A = CD14, B = CD209. Negative selected monocytes in culture for 5 days with two different concentration of GM-CSF and IL-4. Non-differentiated, 300IU/mL or 500IU/mL. The bars indicate the group mean. (n = 4) * p ≤ 0.05, by the Mann-Whitney test.

After determining the culture medium and serum to be used in the procedure for differentiating moDCs, we compared two cytokines concentrations, 300 or 500 UI/mL of rhGM-CSF and rhIL-4. These cytokines concentrations are routinely used in several published protocols to generate moDCs [10]. Moreover, distinct cell culture systems using either plates or tubes were also employed since previous data showed differences in moDC phenotyping based on host cell adhesion [5]. The results are presented in Fig 4 and S2 Table.

First, we observed that in all conditions, tubes or plates with both 300 and 500IU/mL concentrations, a significant difference in the expression of CD14 (Fig 4A and S2 Table) and CD209 (Fig 4B and S2 Table) between non-differentiated and differentiated cells, showing that moDC differentiation occurred. In plates, we observed a higher increase in CD209 levels (300IU/mL 89.1±10.3 and 500IU/mL 88.6±10.1), with a concomitant reduction of CD14 expression (300IU/mL 15.1±26.4 and 500IU/mL 12.16±21.1), regardless of cytokine concentration, when compared with cells cultured in tubes (CD209: 300IU/mL 66.0±26.3 and 500IU/mL 77.1±14.4; CD14: 300IU/mL 30.7±28.6 and 500IU/mL 28.3±21.1). These findings suggest that, under our experimental conditions, moDCs differentiated in plates slightly better when compared to tubes, once considering the expression of the specific moDC surface marker (CD209) and the loss of CD14. Most of the cells from different donors were responsive to both cytokine concentrations, based on an increase in CD209 expression and a concomitant decrease in CD14 levels. However, one donor showed a slight reduction in CD14 (300IU/mL 54.6% *vs* 500IU/mL 43.7%) (S1 Fig).

**Fig 5:**
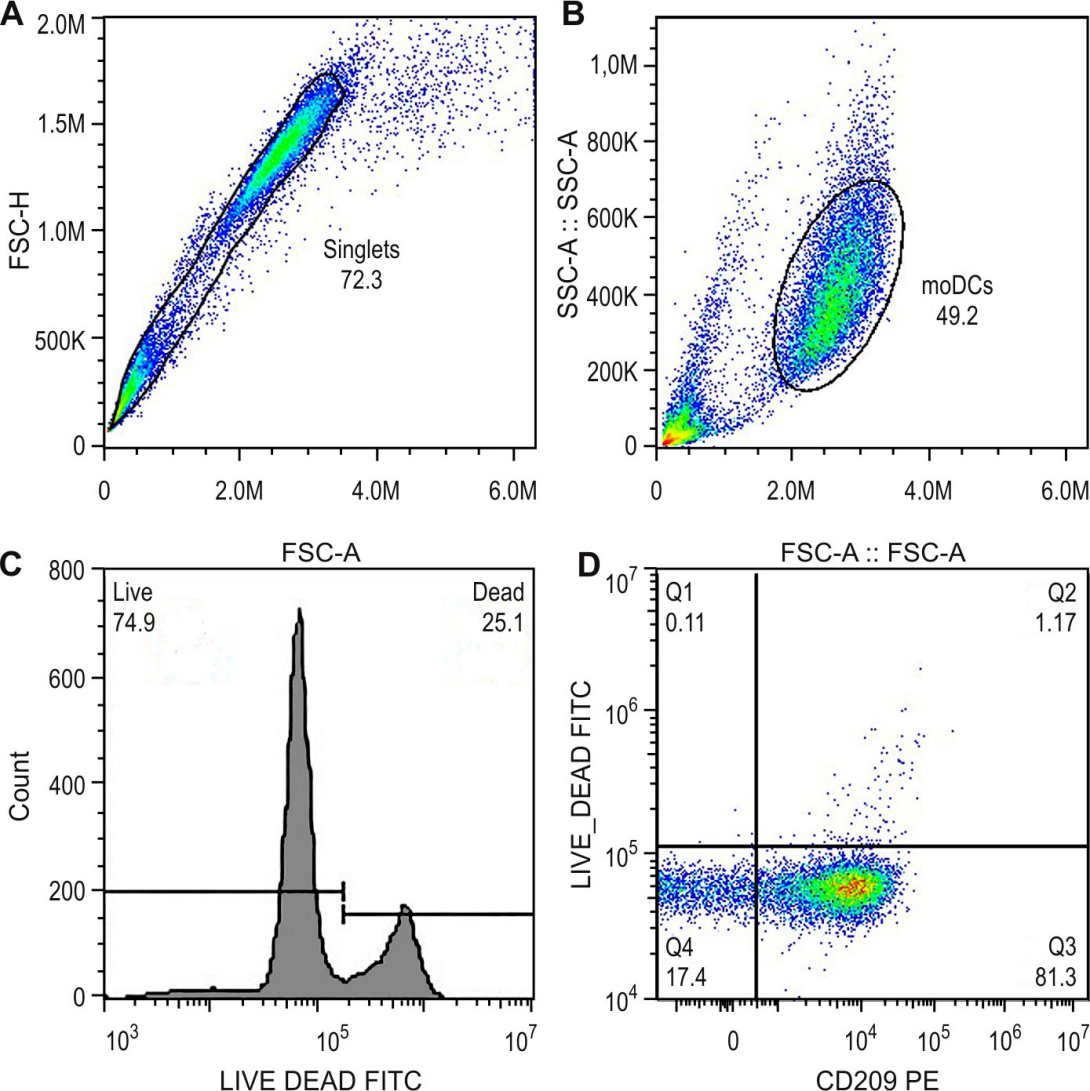
Viability of moDCs after 5 days of culture differentiation. A = FSC-A *vs* FSC-H dotplot for doublets exclusion, gate on singlets. B = dotplot of size (FSC-A) *vs* granularity (SSC-A) for moDCs, inside singlets gate. C = histogram of Viability staining (Live and Dead-FITC) of death control D = Dotplot CD209 *vs* Live and Dead, inside moDCs gate, where Q1 represents CD209^-^ Dead cells (0.11%), Q2 represents CD209^+^ Dead cells (1.17%) Q3 represents CD209^+^ Live cells (81.3%) and Q4 represent CD209^-^ Live cells (17.4%). Graphs representative for cell cultivated in plates with 500IU/mL of cytokines.

After 5 days of culture with cytokines, we observed that our procedure did not affect the cell viability of moDCs at both cytokine concentrations tested (Fig 5).

Since the most efficient protocol for monocytes isolation and differentiation was identified, we performed a functional assay to verify the response/maturation of the obtained moDCs towards LPS stimuli. For this purpose, we analyzed the expression surface markers CD80 and CD86 in different conditions: moDCS + LPS or moDCs without stimulation.

**Fig 6:**
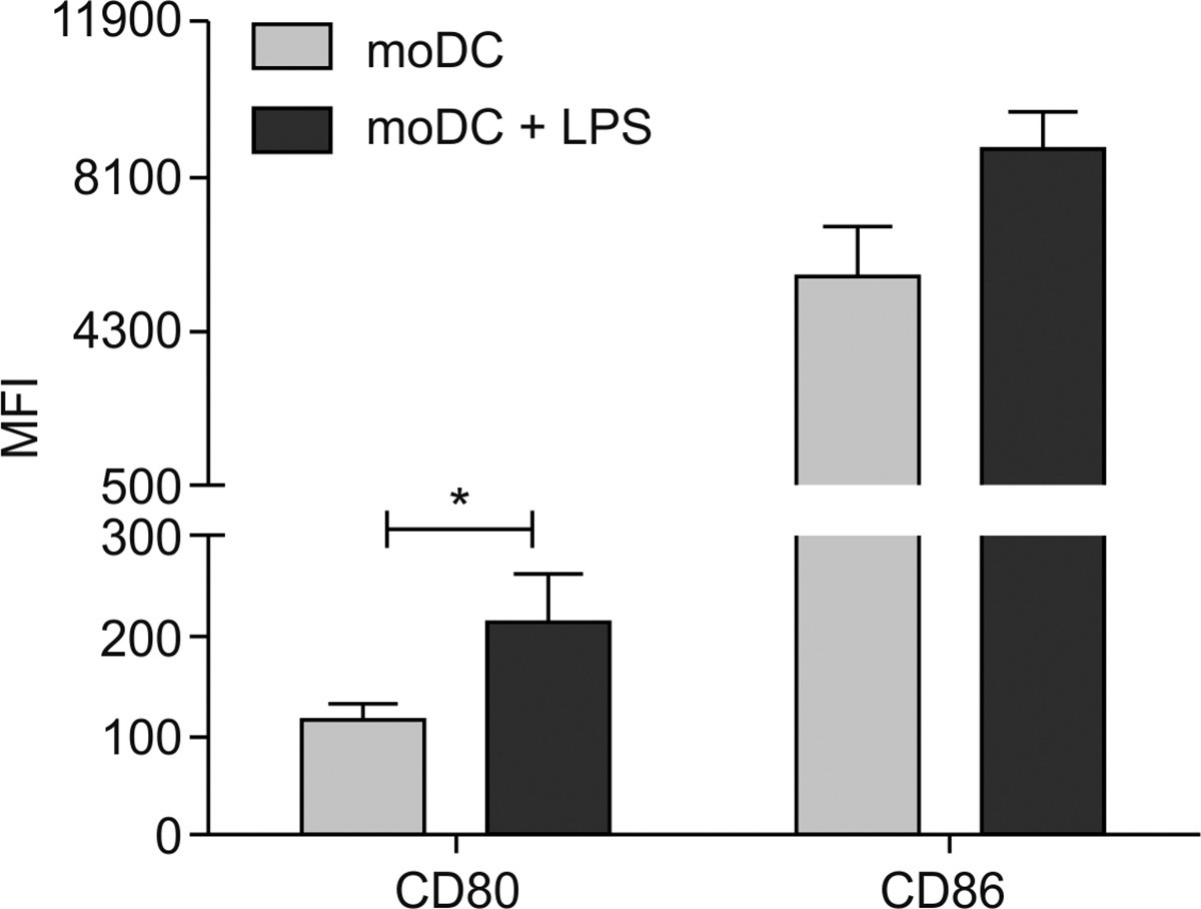
Median Fluorescence Intensity (MFI) of CD80 and CD86. moDC (immature moDCs generated from monocytes by addition of hrGM-CSF and hrIL-4) and moDC + LPS (LPS at 0.5 μg/ml for 24 hours) (n = 8) * p ≤ 0.05, by the Wilcoxon test.

We observed a significant increase in the median fluorescence intensity (MFI) of CD80 in the stimulated cells when compared to the non-stimulated moDCs (p = 0.0547). CD86, however, showed a slight but not significant (p = 0.0625) increase on stimulated moDCs when compared to the cells without stimuli (Fig 6).

## 4. Discussion

Our initial results indicated that despite a significant decrease in lymphocyte contaminants during fresh PBMC purification, the use of in house cold-aggregation followed by self-generating discontinuous Percoll gradient protocol critically affected cell viability, yielding very low rates. This increases the risk of unsuccessful moDC generation. The time consuming nature of the purification process with Percoll further facilitates the cytotoxic effect of this reagent, providing reasons to avoid this step. A previous study demonstrated reduced cell viability when density gradient methods for cell isolation were employed [15].

In order to overcome those limitations and to improve cell viability, the two rounds of cold-aggregation were sequentially used to isolate monocytes directly from PBMC. However, this method was suboptimal as the final product still contained lymphocyte contaminants. Although, Santos et al. (2001) [16] showed modest monocyte isolation using this procedure, the authors did not describe whether lymphocyte contamination was observed in order to make a direct comparison.

Magnetic beads cell-enrichment was a faster and more efficient way to purify monocytes from PBMC. This procedure resulted in high cell viability, and was less laborious compared to the other methods tested. Furthermore, this method has also been validated in other systems [17].

There are two types of magnetic beads isolation, negative and positive selection. There is conflicting evidence as to whether one type of selection is better than the other. Positive selection might affect surface marker expression and cell function. Bhattacharjee and collaborators show that positive selection of CD14^+^ cells results in changes of cell functionality, activation and proliferation when compared to negative selection, and this difference could affect downstream applications [18] such as LPS stimuli or moDCs differentiation. The authors suggested that the temporary binding of CD14 by microbeads could affect cell activation, since this receptor is important in TLR response [18, 19]. Zhou and collaborators also observed that negative selection yields less pure cultures, but they also discussed that, in studies using moDCs a few lymphocyte contaminants were not critical as lymphocytes would not survive more than 1 week in the absence of interleukin 2 (IL-2) [20]. Although both negative and positive selection showed good results, in this study we have chosen negative selection protocol, as we sought to avoid interference in moDC generation. We did obtain a high isolation degree followed by a good quantity in differentiated moDCs with high viability, as demonstrated by our results.

In regards to the culture medium, we have compared RPMI to DMEM. Although no differences in the pattern of expression of CD209 in moDCs was observed, we noticed an increase on the granularity parameter (SSC) of cells once cultivated with RPMI when compared to DMEM. Both DMEM and RPMI media are widely used during ordinary culture procedures of different mammalian cell types [21]. However, according to Wu et al. [19], there are some critical differences in their composition, which might potentially affect cell proliferation, viability, and differentiation. Alternatively, DMEM is supplemented with nutrients, and has different characteristics [21]. Regarding moDC differentiation and cultivation, RPMI medium is the most widely used cell culture medium for that purpose [22]. The supplementation with FBS or HS did not alter the differentiation of cells, under our experimental conditions. Hence, we elected HS as the supplementation serum, since we worked with human cells.

Differentiating cells in plates was slightly more efficient when compared to tubes and although the cytokines concentration of 300IU/mL generated good differentiation for most donors, in one of them the loss of CD14 was less pronounced. These findings indicated that moDCs differentiated better in plates with a final cytokine concentration of 500IU/mL, under our experimental conditions.

To confirm the functionality of differentiated moDCs, we tested the capacity of cells to mature toward LPS stimuli on an assay described elsewhere [2] and comparatively measured the expression of CD80 and CD86 [1]. CD80 and CD86 are expressed on antigen-presenting cells (APCs) and they are up-regulated upon stimulation and activation of APCs [23]. The increase in their expression has been recognized as a common event during DCs maturation [1]. We demonstrated a clear cut enhancement on the surface membrane expression of CD86 and CD80 in moDCs after LPS stimulation, similar to what other studies have previously described [1, 2]. Our study has demonstrated that, the cells isolated and differentiated were able to respond to LPS stimulation by increasing the expression of molecules associated with their maturation. Furthermore, the lower increase of CD86 when compared to CD80 on stimulated moDCs, may be explained by the fact that CD86 is usually constitutively expressed while CD80 is absent or poorly expressed on unstimulated cells [23, 24].

## 5. Conclusions

In the present study, we identified the crucial requirements of the method routinely used for monocyte isolation and subsequent moDC generation. Magnetic-beads isolation of monocytes was more efficient than other techniques, demonstrating an easy, superior, and rapid final result. In addition, cells cultured in RPMI showed better differentiation compared to DMEM, regardless of the serum source. In addition, 500UI/mL of hrGM-CSF and hrIL-4 in combination to a semi-adherent culturing system was the most effective. Interestingly, one out of four donors displayed better moDC differentiation with the highest GM-CSF and IL-4 concentrations. Finally, moDCs cultures using this method are capable of responding to LPS stimulation by increasing expression of CD80 and CD86. These findings shed light on the potential conditions for moDC differentiation protocols, and highlight that further studies are still warranted.

## Supporting information

S1 Table Comparison of different techniques to monocytes purification in human Peripheral Blood Mononuclear Cells (PBMC). (DOCX)

S2 Table The moDCs and non-differentiated monocytes expressing CD14 and CD209 (n = 4). (DOC)

S1 Fig Undifferentiated monocytes (A) and differentiated monocytes in DCs from a poorly responsive donor using two distinct concentrations of GM-CSF and IL-4: 300 IU / ml (B) and 500 IU / ml (C) cultivated in tube. Quadrant Q1 represents CD14 positive, CD14 positive double Q2 and CD209 positive, CD209 positive Q3 and double negative Q4 positive cells, and the number below the quadrant name represents the percentage of positive cells.
(TIF)

S1 Data set (ZIP)
