# Supplementary material for: A protocol for rapid monocyte isolation and generation of singular human monocyte-derived dendritic cells
Source: PLoS One. 2020 Apr 9;15(4):e0231132. doi: 10.1371/journal.pone.0231132 (PMC7145147; doi:10.1371/journal.pone.0231132)
Supplement: S1 Table — (DOCX) [file pone.0231132.s001.docx]

|  | **Lymphocytes** | | **Monocytes** | |
| --- | --- | --- | --- | --- |
|  | **PBMC** | **After isolation** | **PBMC** | **After isolation** |
| **After two cold-aggregation- step (a) + (b)** | 62.1 ± 4.2% | 63.8 ± 9.9% | 5.8 ± 1.2% | 4.6 ± 0.7% |
| **After cold-aggregation + Percoll- step (a) + (c)** | 61.7 ± 5.3% | 21.7 ± 10.8%* | 6.8 ± 2.2% | 48.4 ± 17.7%* |
| **After negative magnetic bead cell-enrichment** | 75.1 ± 8.5% | 8.3 ± 5.2%* | 22.7 ± 8.6% | 89.0 ± 5.1%* |
| **After positive magnetic bead cell-enrichment** | 56.7 ± 17.2% | 3.0 ± 1.8%** | 9.4 ± 3.0% | 79.9 ± 4.9%** |

**S1 Table. Comparison of different techniques to monocytes purification in human peripheral blood mononuclear cells (PBMC).** First line: two consecutive steps of in house cold aggregation (n = 3). Second line: in house cold aggregation plus self-generating discontinuous Percoll gradient (n = 6). Third line: negative magnetic bead cell-enrichment (n = 10). Fourth line: Positive magnetic bead cell-enrichment (n = 5). For step (a) + (b) and step (a) + (c) the monocytes and lymphocytes frequencies were determined by the expression of CD14 and CD3, respectively, and for both magnetic bead cell- enrichment the monocytes and lymphocytes frequencies were determined by morphological parameters (FSC and SSC gating). * p ≤ 0.05 and * p ≤ 0.01, by the Wilcoxon test
