# Supplementary material for: A protocol for rapid monocyte isolation and generation of singular human monocyte-derived dendritic cells
Source: PLoS One. 2020 Apr 9;15(4):e0231132. doi: 10.1371/journal.pone.0231132 (PMC7145147; doi:10.1371/journal.pone.0231132)
Supplement: S2 Table — (DOC) [file pone.0231132.s002.doc]

|  | Non differentiated | 500IU/mL |
| --- | --- | --- |
| CD14 | 86.0% ± 9.3 | 7.6% ± 5.3 ⃰ |
| CD209 | 2.7% ± 2.5 | 84.5% ± 9.3 ⃰ |

Table S2: The moDCs and non-differentiated monocytes expressing CD14 and CD209 (n = 4). Positive selected monocytes cultured in tubes for five days with GM-CSF and IL-4. **Data shown as mean** ± SD. **⃰ p ≤ 0.05, according to the Mann Whitney U test when compared to non differentiated cells.**
