# Supplementary figures and images for: A protocol for rapid monocyte isolation and generation of singular human monocyte-derived dendritic cells
Source: PLoS One. 2020 Apr 9;15(4):e0231132. doi: 10.1371/journal.pone.0231132 (PMC7145147; doi:10.1371/journal.pone.0231132)

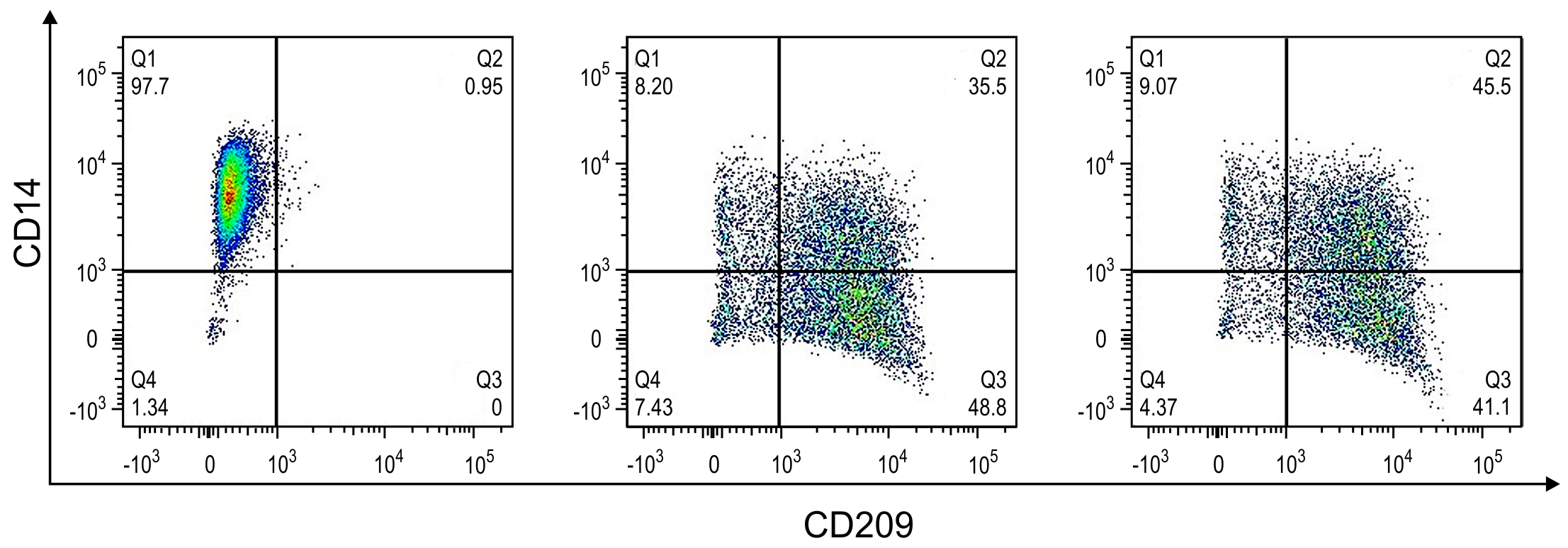

Supplement: S1 Fig — Undifferentiated monocytes (A) and differentiated monocytes in DCs from a poorly responsive donor using two distinct concentrations of GM-CSF and IL-4: 300 IU / ml (B) and 500 IU / ml (C) cultivated in tube. Quadrant Q1 represents CD14 positive, CD14 positive double Q2 and CD209 positive, CD209 positive Q3 and double negative Q4 positive cells, and the number below the quadrant name represents the percentage of positive cells. (TIF) [file pone.0231132.s003.tif]
