# Supplementary material for: A protocol for rapid monocyte isolation and generation of singular human monocyte-derived dendritic cells
Source: PLoS One. 2020 Apr 9;15(4):e0231132. doi: 10.1371/journal.pone.0231132 (PMC7145147; doi:10.1371/journal.pone.0231132)
Supplement: S1 Data set — (ZIP) [file pone.0231132.s004.zip › Magnetic beads isolation.pdf]

Magnetic beads

- Gate strategy: Monocytes and Lymphocytes gate are inside doublets exclusion and PBMC gate, respectively.

PBMC (before magnetic isolation) Monocytes (after magnetic isolation)

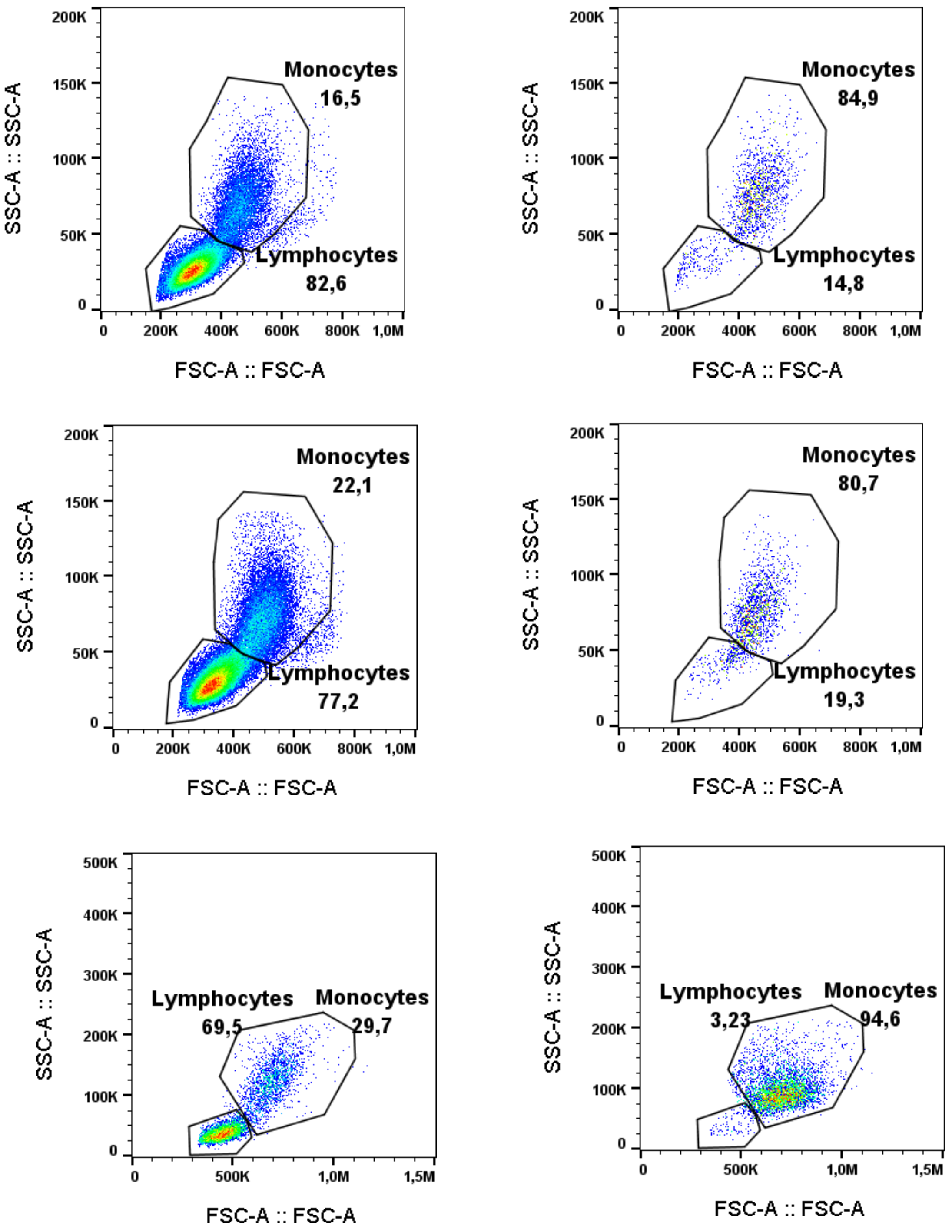

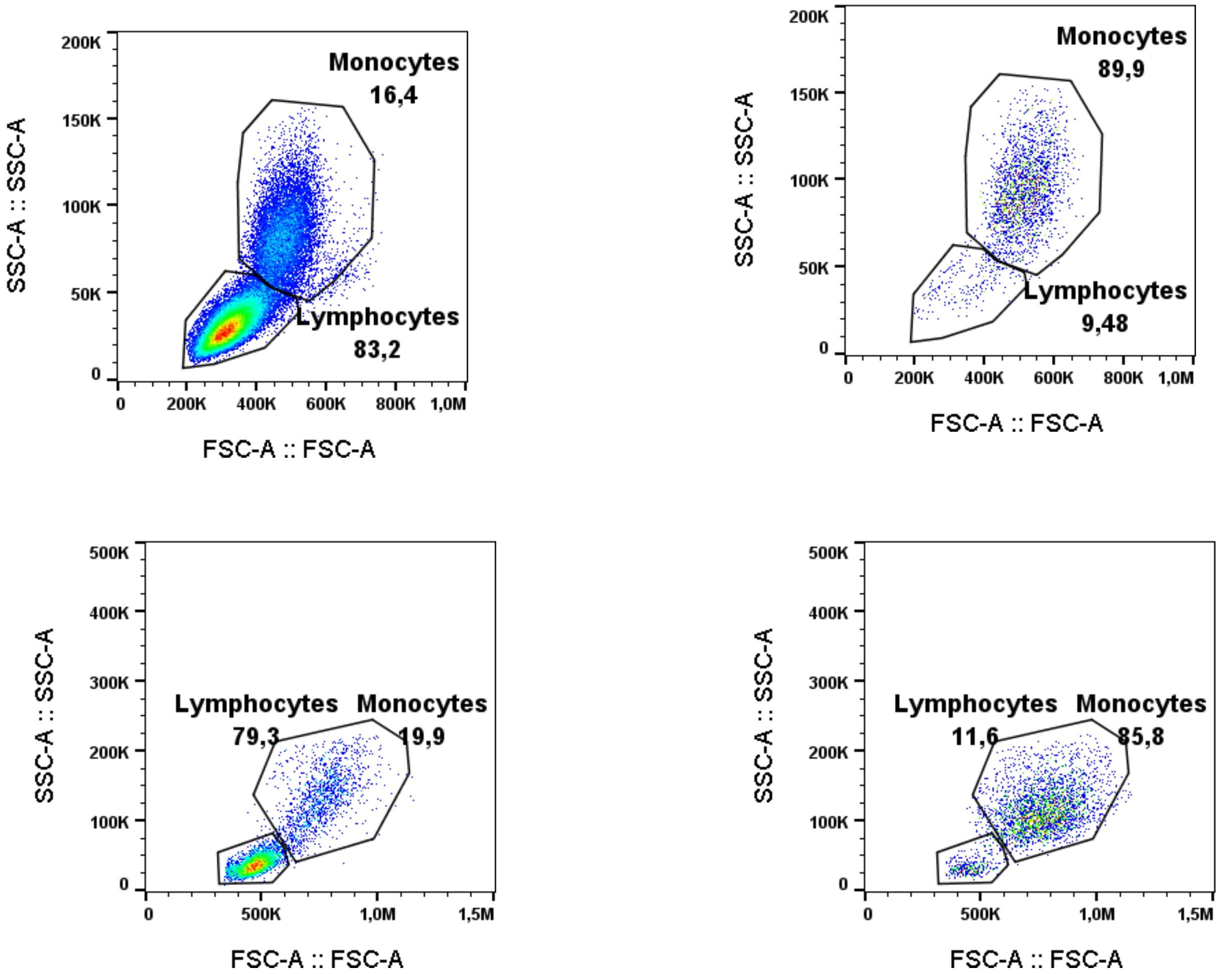
Monocytes
16,4
Lymphocytes
83,2
SSC-A :: SSC-A
FSC-A :: FSC-A
200K
150K
100K
50K
0
200K
400K
600K
800K
1,0M
Monocytes
89,9
Lymphocytes
9,48
SSC-A :: SSC-A
FSC-A :: FSC-A
Lymphocytes
79,3
Monocytes
19,9
500K
400K
300K
200K
100K
0
500K
1,0M
1,5M
SSC-A :: SSC-A
FSC-A :: FSC-A
Lymphocytes
11,6
Monocytes
85,8
SSC-A :: SSC-A
FSC-A :: FSC-A
